# Evaluation of the Anti-Alzheimer Activity of *Lycium barbarum* Polysaccharide in Aβ_1–42_-Induced Neurotoxicity in Rat Model

**DOI:** 10.3390/cimb47040226

**Published:** 2025-03-26

**Authors:** Qingxin Lu, Yixin Meng, Haichi Feng, Xin Di, Xiaoli Guo

**Affiliations:** 1 College of Pharmacy, Ningxia Medical University, Yinchuan 750004, China; lyyjdyxl@163.com (Q.L.); 15147736076@163.com (Y.M.); fhcc918@163.com (H.F.); 2 Key Laboratory of Protection, Development and Utilization of Medicinal Resources in Liupanshan Area, Ningxia Medical University, Ministry of Education, Yinchuan 750004, China; 3 Key Laboratory of Ningxia Minority Medicine Modernization, Ningxia Medical University, Ministry of Education, Yinchuan 750004, China

**Keywords:** Alzheimer’s disease, *Lycium barbarum* polysaccharides, beta-amyloid (_1–42_), oxidative/nitrosative stress, inflammation

## Abstract

As a common neurodegenerative disorder, Alzheimer’s disease (AD) manifests as progressive memory loss, cognitive deficits, and dementia in older adults. As the basis of the traditional Chinese medicinal herb Goji berries, *Lycium barbarum* polysaccharide (LBP) has been proven to exhibit multiple pharmacological activities, including antioxidant, neuroprotective, and anti-inflammatory effects. Evidence supports that LBP can enhance cognitive function and holds promise in counteracting AD. In order to determine the neuroprotective effects of LBP, this study was conducted on an AD rat model induced by intracerebroventricular injection of Aβ_1–42_ peptides. From 24 h after induction until the end of the behavioral experiment, rats were orally administered LBP (150 and 300 mg/kg) once a day. Neurobehavioral parameters were evaluated starting 1 week after administration. After behavioral tests, rats were euthanized, and the whole brain and cortex were isolated to detect the variations in histopathology and biochemical parameters. LBP significantly reversed cognitive impairments, assessed through the Y-maze, Passive Avoidance Test (PAT), and Morris water maze (MWM) test, respectively. Furthermore, LBP not only attenuated NFκB, TNF-α, IL-1β, IL-6, AChE, and oxidative/nitrosative stress levels but also increased IL-4, IL-10, and ACh levels and ChAT activity in the cortex. HE staining also exhibited the neuroprotection of LBP. Our study findings imply that LBP may improve cognitive function through multiple mechanisms and is a potential anti-AD compound.

## 1. Introduction

As an incurable and advancing neurodegenerative disorder, Alzheimer’s disease (AD) is marked by impairments in learning and memory, behavioral abnormalities, an inability to take care of oneself in daily life, and eventually heading toward death [1,2]. The extracellular deposition of senile plaques with β-amyloid (Aβ) as the core, intracellular deposition of neurofibrillary tangles [3], and neuronal and synaptic loss are the main pathological characteristics of AD [4,5]. Moreover, the intra- and extracellular fibrillar aggregates lead to downstream pathologies associated with the severity of cognitive impairments in AD patients, including cholinergic system dysfunction, oxidative stress, inflammation, and neuronal death [6,7,8].

In the past 40 years, multitudinous scientific studies have shown that cholinergic neurons are involved in memory and learning processes. The early loss of them in the AD brain leads to cognitive impairment [9]. The cholinergic system includes the neurotransmitter acetylcholine (ACh), enzymes that c (choline acetyltransferase, ChAT) and degrade (acetylcholinesterase, AChE) ACh, and muscarinic and nicotinic receptors [10]. Extensive clinical evidence demonstrates that the brains of AD patients exhibit a significant loss of cholinergic neurons and ChAT activity, along with a marked deficiency in acetylcholine [11]. Accordingly, restoring the cholinergic system is one of the important clinical therapies for AD treatment. Besides, oxidative stress and neuroinflammation could also contribute to the occurrence and progression of AD [12,13]. The increase of Aβ levels in the brain not only activates the microglia but also directly produces reactive oxygen species (ROS) by binding to the mitochondrial membrane and then causing oxidative stress [14]. This condition arises when the output of ROS exceeds the antioxidant defense capacity [15]. Numerous clinical studies have indicated that there is an increase in ROS and oxidative stress markers in AD patients’ brains [16]. In the AD brain, the overactivated microglia produce a neurotoxic effect by releasing pro-inflammatory cytokines. Studies have demonstrated that inflammatory responses are intimately associated with neuronal pathology and behavioral impairments in AD [17,18]. Therefore, agents possessing antioxidant and anti-inflammatory properties may offer significant benefits in AD [19,20].

As a traditional Chinese medicine, *Lycium barbarum* (*L. barbarum*) L. is mainly composed of multiple components, including polysaccharides, taurine, cerebroside, vitamins, betaine, and flavonoids [21,22]. Among these components, L. barbarum polysaccharide (LBP) is considered a highly valuable and researched constituent. It has been shown that LBP has multiple pharmacological effects, such as anti-aging, antitumor, immunomodulatory, and antioxidant properties [23,24,25,26]. Meanwhile, LBP has also been proven to have sound neuroprotective effects: it could not only improve the impairments of memory and neurogenesis in rats induced by scopolamine [27] and reduce glutamate excitotoxicity in rat cortical neurons [28], but also enhance cellular immunity [29]. Additionally, LBP can also ameliorate pathological events, including neuroinflammation, oxidative stress, and cell apoptosis [30]. This study reveals that oral administration of LBP improves cognitive performance, diminishes Aβ pathology, alleviates oxidative stress and neuroinflammation, restores the cholinergic system, and protects neurons in AD rats. Therefore, LBP holds promise as a multitarget therapeutic agent for AD.

## 2. Materials and Methods

### 2.1. Drugs and Agents

The Aβ_1–42_ peptide (SCP0038; Sigma Aldrich, St. Louis, MO, USA) was mixed in physiological saline (used as a vehicle solution) and then incubated at 37 °C for 7 days to obtain fibrous peptides with neurotoxicity before intracerebroventricular (ICV) injection [25]. LBP, the purity of which is ≥90% [31], was provided by Shanghai Yuanye Biotechnology Co., Ltd. (Shanghai, China) and dissolved in deionized water. Donepezil (Weicai Pharmaceutical Co., Ltd., Suzhou, China) was purchased from the local pharmacy and dissolved in 0.5% CMC-Na.

### 2.2. Experimental Animals and Surgery

The study utilized sixty adult Sprague-Dawley (SD) rats (250–300 g) purchased from the Laboratory Animal Center at Ningxia Medical University, China (License No.: SCXK(Ning) 2020–0001). The 60 rats were housed in specific pathogen-free (SPF)-grade facilities under standardized conditions (23 ± 1 °C; 55 ± 5% humidity). The air supply system was equipped with high-efficiency particulate air (HEPA)-filtered ventilation. Following 7 days of acclimatization, the 60 rats (equal numbers of males and females) were divided into five groups randomly: sham, model (Aβ_1–42_), LBP low-dose (Aβ_1–42_ + 150 mg/kg LBP), LBP high-dose (Aβ_1–42_ + 300 mg/kg LBP), and Donepezil (Aβ_1–42_ + 0.9 mg/kg Donepezil). Following anesthesia with pentobarbital sodium (30 mg/kg), stereotaxic injections were performed to target the lateral ventricle in rats. The coordinates for injection (AP: −1 mm, ML: 1.5 mm, DV: −3.5 mm) were determined concerning the “Rat Brain Stereotaxic Atlas”. The sham group received bilateral intracerebroventricular injections of 10 mM sterile PBS (pH 7.4), while other groups were injected with 5 μL of aggregated Aβ_1–42_ solution. During the operation, a constant rate of injection was maintained to ensure complete absorption of the drug. Starting the day after injection, treatment groups received daily gavage treatment, and the sham group received deionized water at the same volume. Behavioral experiments were conducted after one week of administration. All 12 rats in each group underwent behavioral assessments. Following euthanasia, fresh cortical tissues from 6 animals of each group (3 females and 3 males) were collected for the detection of inflammatory cytokines and oxidative stress markers. The remaining 6 brains (3 females and 3 males) were fixed with paraformaldehyde and then made into paraffin sections for HE staining.

### 2.3. Open Field Test (OFT)

The experimental setup is an open-field box with a high-definition camera mounted above it. During the test, the surrounding environment should be kept quiet. The rats were carefully positioned in the box and left to explore for 5 min without interference. The mean velocity of the rats, the total activity time, and the number of spontaneous activities were recorded.

### 2.4. Morris Water Maze (MWM)

This experimental setup featured a circular tank (160 cm in diameter, 60 cm in height) surrounded by fabric to minimize environmental interference. The circular water tank was equally divided into four quadrants (I, II, III, and IV). Besides, a detachable safety platform (10 cm in diameter) was concealed underwater in quadrant IV. The water temperature was stabilized at 22 ± 1 °C during the trials. The training phase was performed for 6 consecutive days, with two daily training sessions with an interval of 8 h. During the training phase, the rats were gently placed into the water facing the wall of the tank. Rats that successfully reached the platform within 90 s remained on it for 10 s, while those that failed were guided by the experimenter and allowed to stay for the same duration. The reduced latency in reaching the safety platform was used to measure the learning process during the navigation experiment. A probe trial was conducted on the 7th day, with the safety platform removed and each rat undergoing a single trial lasting 90 s. The complete swimming paths of the rats were tracked by a computerized video tracking system (Maze Router V3.1, Techniq Azma Co, Tabriz, Iran).

### 2.5. Y-Maze

The maze comprises three identical plastic arms, each positioned at a 120-degree angle from the others. The maze was wiped with alcohol to eliminate olfactory cues. Rats prefer to visit the arm with less access, thus reflecting their memory of the arms. In a quiet environment, rats were subsequently permitted to explore the maze for 5 min. During this period, the sequence and frequency of arm entries were documented. An entry was registered when four limbs of the rats crossed the inner boundary.

### 2.6. Passive Avoidance Test (PAT)

The setup included dark and light chambers (30 cm × 23 cm × 23 cm) connected by an opening (8 cm × 8 cm). The floor was constructed of copper bars capable of delivering an electric current. In the training phase, the rat was introduced into the light chamber and given 3 min to explore both areas freely. A mild foot shock (0.4 mA, 2 s) was delivered promptly upon entry into the dark chamber. Rats with intact cognitive abilities avoid re-entering the dark chamber due to the associated shock. After 24 h (test session), the rat was reintroduced to the light chamber under the same conditions, but no shock was administered in the dark chamber. The number of times it entered the dark compartment (the number of errors) and when it first entered the dark compartment from the light (the step-through latency, s) within 5 min were recorded and used as the memory score.

### 2.7. Tissue Preparation

Rats were anesthetized via sodium pentobarbital (30 mg/kg, i.p.) after behavioral tests [32]. For six rats in each group, transcardial perfusion was conducted using ice-cold saline to clear the blood, followed by 4% paraformaldehyde to fix the brain tissue. The brains were carefully removed, post-fixed in 4% paraformaldehyde at 4 °C, dehydrated, and embedded in paraffin. Coronal sections were then cut using a microtome (Leica, Wetzlar, Germany) at a thickness of 5 μm. The brains of the remaining rats were preserved at −80 °C for subsequent experiments.

### 2.8. Hematoxylin-Eosin Staining (HE)

To prepare the brain sections for staining, sections were deparaffinized with xylene and rehydrated through ethanol. Subsequently, hematoxylin-eosin staining was applied to counterstain the tissues. Finally, the brain tissue morphology changes were examined using an optical microscope at 40× magnification.

### 2.9. Detection of Brain Inflammation Markers

To determine the levels of IL-1β, IL-4, IL-6, IL-10, NFκB, and TNF-α, ELISA kits (Nanjing Jiancheng Bioengineering Institute, Nanjing, China) were utilized. The cortical tissues of rat brains were homogenized in a buffer according to the instructions. Following centrifugation (10,000 rpm, 20 min) at 4 °C, the supernatants were analyzed for cytokine concentrations using ELISA, with quantification performed via a plate reader (Thermo Fisher Scientific, Waltham, MA, USA).

### 2.10. Detection of Biomarkers for Oxidative Stress and Cholinergic System

Following the protocol of the biochemical markers kit, the supernatant of cerebral cortex tissues from the rats was collected to detect the levels of MDA, ROS, NO, and ACh, and the activity of CAT, SOD, GSH-Px, NOS, AChE, and ChAT, using kits provided by Nanjing Jiancheng Bioengineering Institute, China.

### 2.11. Statistical Analysis

Data are presented as mean ± standard deviation (SD). Prior to the main analysis, data were tested for normality using the Shapiro-Wilk test and for homogeneity of variances using Levene’s test, respectively. Statistical analyses were performed using one-way analysis of variance (ANOVA) in SPSS 21.0. When the ANOVA indicated a significant difference among groups, post-hoc analyses were conducted using Fisher’s Least Significant Difference (LSD) test to determine which specific groups differed from each other. Statistical significance was defined as *p* < 0.05.

## 3. Results

### 3.1. LBP Mitigates Memory Impairments Caused by Aβ_1–42_ Peptides

**Figure 1:**
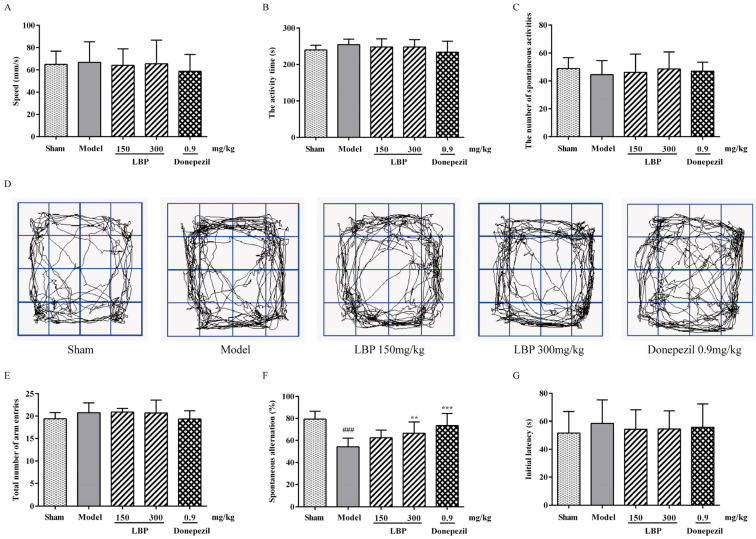

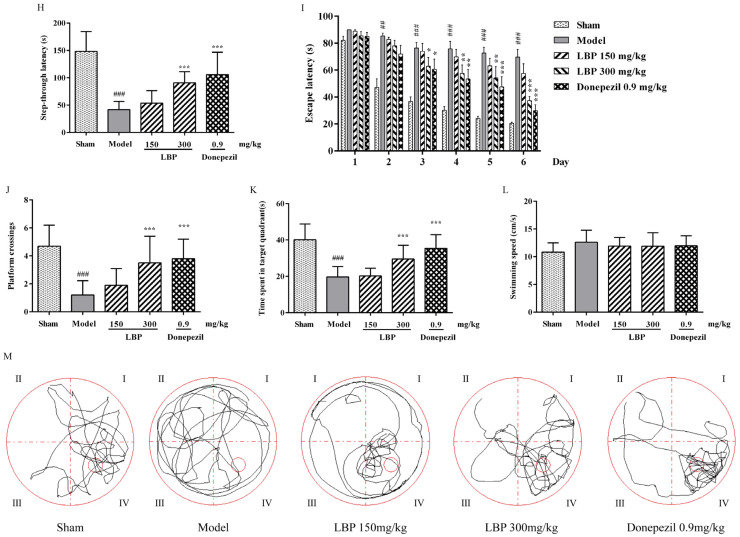
LBP notably enhanced the spontaneous alternation behavior in Aβ_1–42_-induced rats. (**A**–**C**) The speed, activity time, and the number of spontaneous activities of the rats in the OFT. (**D**) The video track of the OFT. (**E**,**F**) The total number of arm entries and the spontaneous alternation (%) in the Y-maze test. (**G**,**H**) The initial latency and the step-through latency in the PAT. (**I**) The escape latency of rats seeking water during the training phase of the MWM test. (**J**,**K**) The platform crossings and the time spent in the target quadrant of rats during the probe trial of the MWM test. (**L**) The swimming speed among the study groups in the MWM test. (**M**) Represents the video trajectory during the probe trial. The data are presented as the mean ± SD, *n* = 12, ^##^ *p* < 0.01 and ^###^ *p* < 0.001 compared with the sham group; * *p* < 0.05, ** *p* < 0.01, and *** *p* < 0.001 compared with the Aβ_1–42_ group.

First, the OFT was used to detect if there was interference of locomotor deficits in memory tasks. As depicted in Figure 1A–D, no significant differences were observed in mean velocity, total activity time, or the number of spontaneous activities across all groups, eliminating the possibility of motor deficits interfering with memory tasks. In the Y-maze experiment, there were no significant differences in the total number of arm entries across the groups (Figure 1E), suggesting that the rats’ motor and exploratory functions were unaffected by olfactory stimulation. Treatment with Aβ_1–42_ resulted in a considerable decrease in the percentage of spontaneous alternation in the model group relative to the sham group, which was increased after LBP treatment (Figure 1F). Thereafter, the role of LBP in the long-term memory of rats treated with Aβ_1–42_ was examined using PAT and MWM tests. In the acquisition phase of the PAT test, no significant differences in initial latency were observed across all groups (Figure 1G). On the contrary, the step-through latency of Aβ_1–42_-treated rats was significantly shorter than that of the sham rats in the retention test. Additionally, the LBP-treated rats demonstrated longer step-through latency than the Aβ_1–42_-treated rats (Figure 1H). During the training phase of the MWM test, the ICV- Aβ_1–42_ peptides significantly prolonged the model group’s escape latency over several consecutive days, indicating impaired memory function. In contrast, treatment with LBP at 300 mg/kg doses remarkably shortened escape latency in model rats, which depicted the memory-improving effect of LBP (Figure 1I). In the probe test, LBP-treated rats had more crossings of the platform and longer swimming time in the target quadrant than AD rats (Figure 1J,K). Nevertheless, the swimming speed across the experimental groups showed no significant difference (Figure 1L). The swim trajectory plot (Figure 1M) demonstrates the aforementioned behavioral alterations.

### 3.2. LBP Alleviates Oxidative/Nitrosative Stress Markers in the Brain of AD Rats

**Figure 2:**
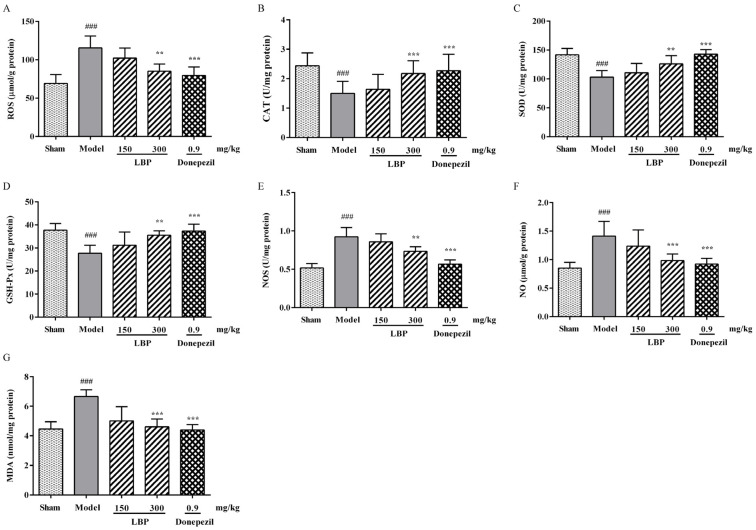
Effects of LBP on oxidative-antioxidative status in the cerebral cortex of Aβ_1–42_-induced rats, including the levels of ROS (**A**), NO (**F**), and MDA (**G**) and the activities of CAT (**B**), SOD (**C**), GSH-Px (**D**), and NOS (**E**). The data are presented as the mean ± SD, *n* = 6, ^###^ *p* < 0.001 compared with the sham group; ** *p* < 0.01 and *** *p* < 0.001 compared with the Aβ_1–42_ group.

Concerning oxidative stress indicators, our data indicated that, in comparison with the sham group, AD rats experienced a substantial rise in ROS levels within the cerebral cortex. However, treatment with LBP (300 mg/kg) effectively mitigated this abnormal increase (Figure 2A). This study unlocked the potential of LBP in tipping the balance of antioxidants (CAT, SOD, and GSH) over the oxidative stressors (NOS, NO, and MDA). As depicted in Figure 2B–E, when compared to the Aβ_1–42_ group, LBP effectively restored the activities of CAT, SOD, and GSH-Px to a significant extent, while also inhibiting NOS activity to a level nearly equivalent to that of the sham group. Also, LBP could decrease the contents of NO and MDA in AD rats’ brains (Figure 2F,G).

### 3.3. LBP Attenuates the Inflammation in Rats’ Brains Induced by Aβ_1–42_

**Figure 3:**
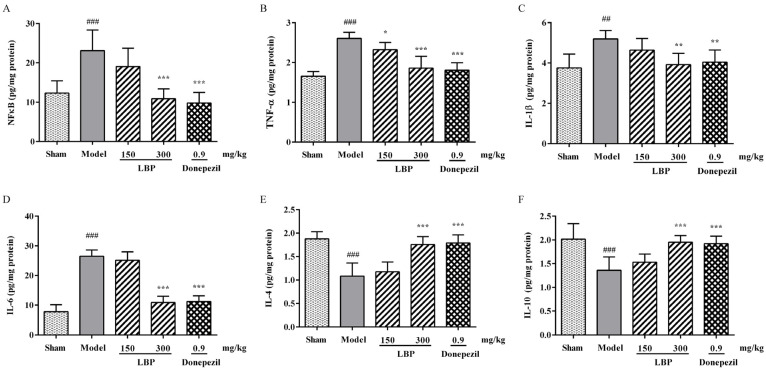
The effect of LBP on the levels of NFκB (**A**), TNF-α (**B**), IL-1β (**C**), IL-6 (**D**), as well as IL-4 (**E**) and IL-10 (**F**) in the cerebral cortex of Aβ_1–42_-induced rats. The data are presented as the mean ± SD, *n* = 6, ^##^ *p* < 0.01 and ^###^ *p* < 0.001 compared with the sham group; * *p* < 0.05, ** *p* < 0.01 and *** *p* < 0.001 compared with the Aβ_1–42_ group.

Neuroinflammatory factors, along with elevated levels of Aβ_1–42_ and oxidative/nitrosative stress markers, also have an important impact on the progression of AD. Therefore, specific ELISA kits were utilized to measure the concentrations of several key neuroinflammatory cytokines in rat brains. As shown in Figure 3, the ICV injection of Aβ_1–42_ peptides was discovered to raise the levels of NFκB, TNF-α, IL-1β, and IL-6 while decreasing the contents of IL-4 and IL-10 in the rat cerebral cortex. The above differences were significant compared to the sham group. Meanwhile, these changes in neuroinflammatory cytokine levels mentioned above were remarkably reversed following LBP treatment.

### 3.4. LBP Protects Cholinergic System from Aβ_1–42_ Peptides

**Figure 4:**
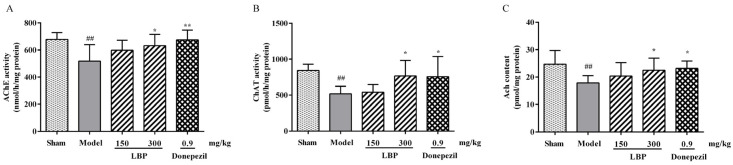
The effect of LBP on the activities of AChE (**A**) and ChAT (**B**), and the level of ACh (**C**) in the cerebral cortex of Aβ_1–42_-induced rats. The data are presented as the mean ± SD, *n* = 6, ^##^ *p* < 0.01 compared with the sham group; * *p* < 0.05 and ** *p* < 0.01 compared with the Aβ_1–42_ group.

The disruption in the cholinergic nervous system has been proven to have a strong connection with cognitive decline and memory loss. The levels of cholinergic neurotransmitters in rats among groups were depicted in Figure 4. In the Aβ_1–42_ group, AChE activity was notably elevated compared to the sham group (Figure 4A), while a significant decrease in ACh levels and ChAT activity was observed (Figure 4B,C), indicating impairment of the brain’s cholinergic system. However, administering LBP can significantly reverse the above results.

### 3.5. LBP Protects Against Neurological Injury in AD Model Rats

**Figure 5:**
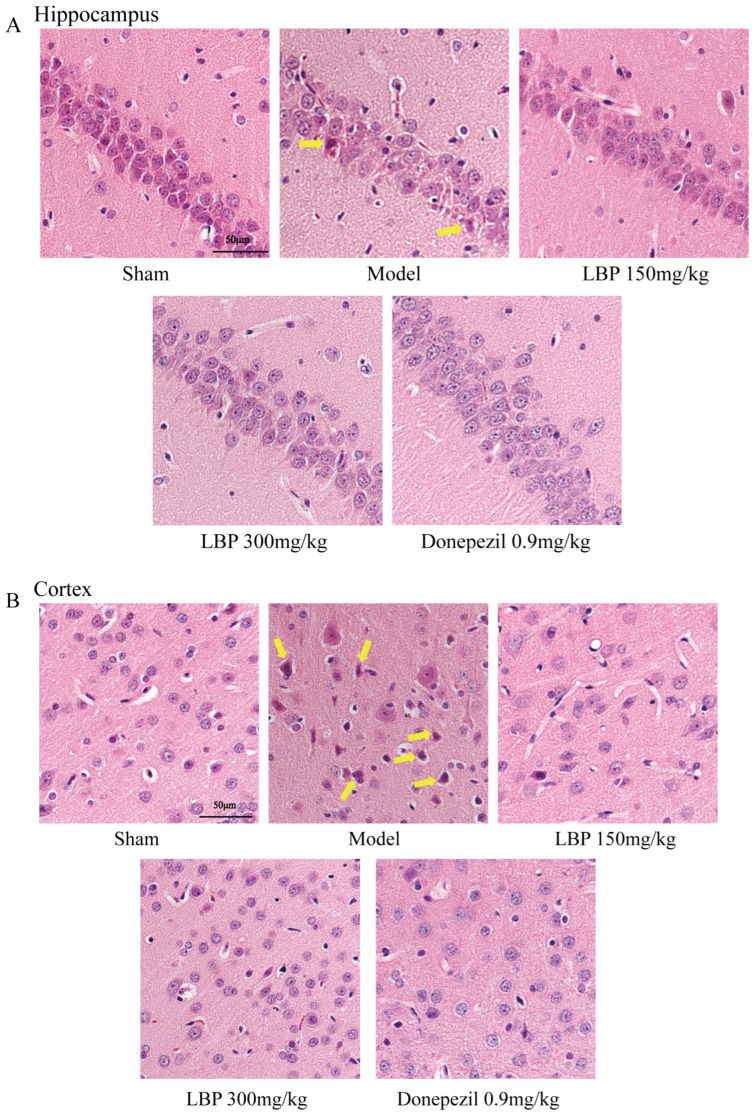
The protective effect of LBP on the hippocampal and cortical neurons in the brains of Aβ_1–42_-induced rats. Histology of CA1 region of hippocampus (**A**) and cortex (**B**) with HE staining at 40× magnification (50 μm). The yellow arrows denote damaged neurons.

Histopathological analysis of hippocampal neuron morphology in each experimental group of rats was implemented using HE staining. As illustrated in Figure 5, the sham group exhibited intact and tightly packed hippocampal neurons with prominent nuclei. However, Aβ_1–42_ infused group exhibits toxic effects including irregularly shaped, and loosely arranged with deformities. At the same time, eosinophilic cytoplasm, karyopyknosis, and vacuolization also significantly increased. In comparison, treatment with LBP demonstrated relatively regular cellular morphology and reduced eosinophilic stained neurons, which represent mild neurotoxicity (Figure 5A). Similarly, cortical neurons in Aβ_1–42_-injection rats’ brains were also protected after treatment with LBP (Figure 5B).

## 4. Discussion

Although several medications, including donepezil and memantine, have been sanctioned by the United States Food and Drug Administration [33,34], the complex pathological pathways of AD limit the efficacy of these drugs. This makes preventing or treating AD full of challenges. Numerous studies have confirmed that Aβ enriches in the hippocampus and cortex first, and then accumulates in the extracellular space to form senile plaques [35]. In addition, it has been confirmed that cholinergic dysfunction, oxidative stress, and neuroinflammation are all involved in the development and progression of AD [36]. In this study, we investigated the effects of LBP in an Aβ_1–42_-induced rat model of AD.

Aβ_1–42_, the most important toxic component of β-amyloid protein, is intimately associated with the onset and progression of AD [37]. Numerous studies have confirmed that intracranial injection of Aβ in rodents induces pathological symptoms, such as obvious cognitive impairment, overexpression of toxic proteins, cholinergic deficiency, oxidative stress, and apoptosis, which are strikingly similar to those of AD patients [38,39]. These studies make intracranial injection of Aβ a suitable experimental model for exploring both the potential pathogenesis mechanisms and therapeutic measures of AD. Here, an AD model was established through ICV injection of Aβ_1–42_ in rats. The results of behavioral experiments indicate that the SD rats exhibited significant cognitive impairment after intraventricular injection of Aβ_1–42_, while LBP treatment improved cognitive function. The results of behavioral experiments indicate that the SD rats exhibited significant cognitive impairment after intraventricular injection of Aβ_1–42_, while LBP treatment improved cognitive function. The data from the open field test confirmed that Aβ_1–42_ infusion and LBP treatment did not cause any changes in the motor ability of rats. Similarly, the absence of significant differences in swimming speed among all groups of rats in the MWM test is also supported the above results. The working memory ability of animals is usually tested by the Y-maze. The LBP treatment improved the working memory ability of the rats, as reflected in the significant improvement of the spontaneous alternation reaction rate. Next, the MWM experiment was conducted to investigate the rats’ learning and spatial memory abilities across groups [40]. LBP administration shortened the escape latency, which indicated that the impaired learning and memory abilities of rats caused by Aβ_1–42_ have been improved to a certain extent. Meanwhile, the increased number of platform crossings and extended time spent in the target quadrant during the probe test phase indicated that LBP administration enhanced spatial memory ability in rats injured by ICV injection of Aβ_1–42_. The PAT is based on passive avoidance responses in fear-induced situations [41]. Many studies have confirmed that avoidance memory deteriorated after injecting Aβ into the intracranial cavity of rodents [42,43]. In our study, LBP administration restored avoidance memory in AD rats after 24 h. Therefore, although intracranial injection of Aβ_1–42_ worsened memory-related behavioral manifestations in rats, LBP treatment improved Aβ_1–42_-related changes in short-term memory, spatial memory, and avoidance memory.

In AD, the critical expansion of nitrite levels contributes to nitrosative stress, which subsequently induces the generation of free NO species, also known as reactive nitrogen species (RNS) [44]. The increased level of RNS facilitates lipid peroxidation and mitochondrial damage, ultimately leading to oxidative stress [45]. Indeed, even in our study, intracranial Aβ_1–42_ injection in rats demonstrated a significant increase in NO and MDA levels and NOS activity, which was consistent with previous research findings [46]. In contrast, LBP treatment altogether significantly attenuated NO and MDA levels and NOS activity following intracranial Aβ_1–42_ injection in rats. As a meaningful indicator of oxidative stress [47], the excessive generation of ROS has been shown to correlate strongly with apoptosis. The increase of ROS may inhibit both synaptic activity and neurotransmitter generation and transmission of neurons by affecting DNA transcription, lipid peroxidation, and mitochondrial functional structure, ultimately resulting in cognitive decline. These metabolic abnormalities can further promote Aβ accumulation and tau hyperphosphorylation, exacerbating ROS production and forming a vicious cycle of apoptosis. The increase of ROS may inhibit both synaptic activity and neurotransmitter generation and transmission of neurons by affecting DNA transcription, lipid peroxidation, and mitochondrial functional structure, ultimately leading to cognitive dysfunction. Such aberrant cellular metabolic conditions can enhance Aβ generation and tau hyperphosphorylation, exacerbating ROS production and forming a vicious cycle [48]. In vivo, SOD, GSH-Px, and CAT together form the first line of defense of the antioxidant defense system, which can counteract the production of ROS during oxidative stress. Superoxide radicals are decomposed by SOD to form hydrogen peroxide, while GSH-Px and CAT further decompose hydrogen peroxide into water and oxygen, thereby suppressing the generation of hydroxyl free radicals [49]. The above three enzymes interact synergistically at different sites in the free radical metabolism pathway. Our further research has found that ROS level was raised significantly after Aβ_1–42_ injection, accompanied by the decreased activities of SOD, CAT, and GSH-Px. Intriguingly, LBP regulated the oxidative and antioxidant status of Aβ_1–42_-treated rats by enhancing activities of SOD, CAT, and GSH-Px, thereby reducing ROS levels, confirming its ability in free radical scavenging and antioxidant activity.

In AD, pathological progression, oxidative stress, and neuroinflammation are intertwined phenomena [12]. Under conditions of neurodegenerative diseases, ROS activates the transcription factor NFκB in glial cells, causing it to transfer from the cytoplasm to the nucleus, thereby promoting the transcription of some important pro-inflammatory genes, including IL-1β, IL-6, and TNF-α [50,51]. Elevated levels of pro-inflammatory cytokines have been detected in both the serum and brain tissue of AD patients [52,53]. Neuroinflammation is harmful to neurogenesis, synaptic function, and neuronal vitality, which are the foundations of cognitive and behavioral functions [17,54]. The neuroinflammation induced by Aβ_1–42_ contributes to the onset and progression of neuronal loss and behavioral and psychological disorders, including decreased spatial cognitive ability, anxiety and depression behaviors, and exploratory/motor disorders [55,56]. The results of this research demonstrated that Aβ_1–42_ injection facilitated the overproduction of inflammatory markers, including NFκB, IL-1β, TNF-α, and IL-6, in the rat cortex, aligning with prior research [46]. Also, the contents of two anti-inflammatory factors, IL-4 and IL-10, are significantly decreased in Aβ_1–42_-induced rats. However, LBP administration inhibited the excessive production of pro-inflammatory factors NFκB, IL-1β, TNF-α, and IL-6, thereby suppressing the neuroinflammatory response of Aβ_1–42_-exposed rats, demonstrating its good anti-inflammatory effect. The aforementioned results indicate that the improvement effect of LBP on cognitive impairment induced by Aβ_1–42_ is at least partially attributed to its anti-inflammatory properties.

The cholinergic system participates in many processes of the nervous system within the body, including learning, memory, and emotions. In AD, cognitive impairment is linked to brain levels of ACh and AChE [11]. ChAT catalyzes the synthesis of ACh from choline and acetyl CoA, which is then released into the synaptic cleft and broken down into acetate and choline by AChE [57]. In this experiment, ICV-Aβ_1–42_ injection significantly elevated AChE activity while reducing ChAT activity in the brains of AD rats, leading to a substantial drop in ACh levels. Administration of LBP reversed the aforementioned changes, indicating that its capacity to mitigate cholinergic dysfunction may contribute to enhanced cognitive performance.

Previous reports have shown that the infusion of Aβ_1–42_ into the lateral ventricle of rats results in cognitive deficits that are associated with neuronal damage. We performed HE staining on the cerebral cortex and hippocampal CA1 region of rats to evaluate both morphological and quantitative changes in the neurons after ICV-Aβ_1–42_ injection, as well as the therapeutic effect of LBP. As shown in the photomicrograph of the HE staining and consistent with previous research that confirmed the ability of ICV-Aβ_1–42_ infusion to induce serious damage to neurons [34,46], the cerebral cortex and hippocampus CA1 region of Aβ_1–42_-injected rats showed extensive neuronal shrinking and neuronal nuclear pyknosis, with some cells undergoing high-density eosinophilic staining of cytoplasm. In the brains of rats treated with 300 mg/kg LBP, we observed more surviving neurons with clear nuclear and cytoplasmic integrity, which confirms the potential of LBP in preventing widespread neuronal death and improving cell survival rates.

Dysfunction of the blood-brain barrier (BBB) triggers neuroinflammation and oxidative stress, which subsequently enhance the activities of β-secretase and γ-secretase, ultimately promoting Aβ production [58]. In addition, dysbiosis of the gut microbiota could stimulate the secretion of Aβ and lipopolysaccharides to impair gastrointestinal permeability and exacerbate neuroinflammation, which then leads to neuronal death in AD patients [59]. Research has confirmed that LBP can improve BBB dysfunction and show the potential of prebiotics [60,61]. In our study, LBP protects neurons in the brains of AD rats by suppressing oxidative stress and alleviating neuroinflammation. Therefore, we speculate that the neuroprotective effects of LBP may be associated with its potential to ameliorate BBB dysfunction and modulate gut microbiota. However, the precise molecular mechanisms underlying these effects require further investigation.

## 5. Conclusions

This study confirmed that LBP administration improved cognitive impairment and reduced neuronal damage induced by ICV-Aβ_1–42_. These effects of LBP were related to improving cholinergic system damage, inhibiting oxidative stress, and reducing neuroinflammation. Our research findings prove that LBP may be a promising compound for treating brain retrogression associated with AD. As it should be, the other mechanisms involved in the neuroprotective effects of LBP should be evaluated through further research.

## Data Availability

The original contributions presented in the study are included in the article. Further inquiries can be directed to the corresponding authors.
